# Bilayer Films of Poly(lactic acid) and Cottonseed Protein for Packaging Applications

**DOI:** 10.3390/polym15061425

**Published:** 2023-03-13

**Authors:** Atanu Biswas, Huai N. Cheng, Gary Kuzniar, Zhongqi He, Sanghoon Kim, Roselayne F. Furtado, Carlucio R. Alves, Brajendra K. Sharma

**Affiliations:** 1 National Center for Agricultural Utilization Research, USDA Agricultural Research Service, Peoria, IL 61604, USA; 2 Southern Regional Research Center, USDA Agricultural Research Service, New Orleans, LA 70124, USA; 3 Embrapa Agroindústria Tropical, Rua Dra. Sara Mesquita 2270, Fortaleza 60511-110, CE, Brazil; 4 Chemistry Department, State University of Ceará, Silas Munguba Av. 1.700, Fortaleza 60740-020, CE, Brazil; 5 Eastern Regional Research Center, USDA Agricultural Research Service, Wyndmoor, PA 19038, USA

**Keywords:** poly(lactic acid), cottonseed meal, cottonseed protein, bilayer film, food packaging

## Abstract

Poly(lactic acid) (PLA) is a common biobased film-former made from renewable biomass, such as polysaccharides from sugarcane, corn, or cassava. It has good physical properties but is relatively expensive when compared to the plastics used for food packaging. In this work, bilayer films were designed, incorporating a PLA layer and a layer of washed cottonseed meal (CSM), an inexpensive agro-based raw material from cotton manufacturing, where the main component is cottonseed protein. These bilayer films were made through the solvent casting method. The combined thickness of the PLA/CSM bilayer film was between 47 and 83 μm. The thickness of the PLA layer in this film was 10%, 30%, or 50% of the total bilayer film’s thickness. Mechanical properties of the films, opacity, water vapor permeation, and thermal properties were evaluated. Since PLA and CSM are both agro-based, sustainable, and biodegradable, the bilayer film may be used as an eco-friendlier food packaging material, which helps reduce the environmental problems of plastic waste and microplastics. Moreover, the utilization of cottonseed meal may add value to this cotton byproduct and provide a potential economic benefit to cotton farmers.

## 1. Introduction

Plastics in packaging have attracted much negative publicity because of the environmental concerns regarding their accumulation in the environment and the effects of microplastics in water on health. One of the solutions to this problem is the use of agro-based materials and biobased plastics [1,2,3,4]. Poly(lactic acid) (PLA) is one of the most commercially successful biobased plastics because of its processability and mechanical properties [5,6,7,8]. It has been shown to be biodegradable [9,10]. The global PLA market size was valued at USD 566.74 million in 2021 and was expected to advance at a compound annual growth rate of over 26.6% from 2022 to 2030 [11]. However, PLA is also relatively expensive relative to traditional petroleum-based plastics [5,8].

Proteins are also among the more commonly used materials for food packaging studies [12,13]. For example, cottonseed protein is obtained from the kernel of cottonseed [14] and has been studied as a bioplastic [15,16,17,18] and as a wood adhesive [14]. It is biodegradable because it is a naturally occurring protein [19]. However, cottonseed protein films are brittle and require use of additives or other treatments to improve their mechanical properties. Some of the treatments include the addition of a plasticizer, a denaturant, or a crosslinker [16,20,21,22]. In a recent study [23], cottonseed protein isolate was produced by extraction of cottonseed meal by base, acid precipitation, washing, centrifuging, and freeze drying; it was then dissolved in formic acid to produce the film, with the addition of six plasticizers (including glycerol and levulinic acid). Films with 5–40% plasticizer were made, and the mechanical properties determined.

As PLA and cottonseed protein are promising bioplastics, it may be desirable to use both materials together for food packaging. In this work, we have made films comprising a bilayer of PLA and washed cottonseed meal (CSM), plasticized with glycerol. In this way, we can take advantage of the beneficial features of both materials and reduce the cost of PLA through the addition of CSM. In previous work [24,25], it has been shown that the inexpensive and commercially available CSM washed with water or buffer contains about 70% protein and is as effective as cottonseed protein isolate in wood adhesive strength.

Whereas the layering technique is known in PLA film-making, only a few papers have previously been reported on bilayer films involving PLA and a protein. For example, two papers appeared on PLA–gelatin bilayer films [26,27], one paper on PLA–gelatin–PLA tri-layer films [28], and one paper on PLA–soy protein bilayer films [29]. In a different study, soy protein films were dipped in PLA solutions and dried [30]. In another study [31], bilayer nanofiber sheets were produced via electrospinning that comprised PLA, soy protein, and hydroxypropyl methylcellulose. As far as we know, the present work represents the first report of PLA–CSM (or cottonseed protein) bilayer films for packaging applications.

## 2. Materials and Methods

### 2.1. Materials

CSM was obtained from Cotton Inc. (Cary, NC, USA) and washed according to previous publications [24,25]. Briefly, the CSM was first ground to less than 0.5 mm in particle size. It was then mixed well with eight times the amount of water for 30 min and then centrifuged for 15 min at 6000 rpm. The resulting solid was then subjected to a second washing with equal weight of water and separated out through centrifugation again at 6000 rpm for 15 min. It was finally dried and milled to obviate the formation of clumps. The protein content of the washed CSM was about 65%. The rest of the material contained mostly carbohydrates with lesser amounts of lignin and inorganics.

PLA was an Ingeo^TM^ 2003D sample obtained from Jamplast, Inc., Ellisville, MO, USA. Glycerol, methylene chloride, and sodium hydroxide were acquired from Sigma-Aldrich, Milwaukee, WI, USA.

### 2.2. Film Preparation

The CSM films were prepared by the “casting” method adapted from previous publications [29,32,33]. For each film, 1 g CSM and 0.3 g glycerol were added into 20 mL distilled water. The pH was adjusted to 10.5 with 2N NaOH. The mixture was heated to 80 °C with stirring for 1 h to dissolve the CSM. The solution was then filtered three times with a coarse filter paper. The filtrate (containing about 1 g of the 1.0:0.3 CSM: glycerol mixture) was then poured into a polypropylene tray and dried at 50 °C to produce the CSM films (with 23% glycerol). The PLA films were prepared by dissolving a specific amount of PLA in 400 mL methylene chloride with stirring. For each film, the solution was gently poured into the dish, and methylene chloride allowed to evaporate at room temperature in a hood. For bilayer CSM/PLA films (with weight ratios of 90:10; 70:30, 50:50), the PLA/methylene chloride solutions (with 0.11 g, 0.43 g, and 1.0 g PLA, respectively) were poured onto the dried CSM film in each of the trays and then dried at room temperature in a hood. Each film was cut into at least 5 equal rectangular strips with the dimensions of 12.5 mm wide and 280 mm long. All the film strips were conditioned in a temperature- and humidity-controlled room (23 ± 1 °C and 50 ± 5% RH) for at least 40 h before mechanical testing.

### 2.3. FT-IR-ATR

For Fourier transform infrared (FT-IR) measurements, a Frontier spectrometer (Perkin Elmer, Waltham, MA, USA) was used in the Attenuated Total Reflectance (ATR) mode, with a diamond ATR crystal. The instrumental conditions included: 650–4000 cm^−1^ spectral region, 16 scans per spectrum, 4 cm^−1^ resolution, and room temperature. Data were transported to a computer and processed with an Excel spreadsheet (Microsoft, Redmond, WA, USA).

### 2.4. Mechanical Properties of the Films

The thicknesses of test specimens were measured at five different locations with an ElectroPhysik minitest (Model No. 3100, Dr. Steingroever GmbH & Co., KG, Cologne, Germany), and the average values were used for the calculations. Young’s modulus (YM), tensile strength (TS), and elongation at break (EB) were measured for each sample using an Instron Universal testing system (Model 3365, Instron Corp., Norwood, MA, USA; Bluehill Universal software). The initial gauge length was set at 250 mm, and the rate of grip separation was 25 mm/min [34], using a 1 kN load cell. Each test was repeated with five film strips, and the result was reported as average ± standard deviation.

### 2.5. Water Vapor Permeability (WVP)

The WVP was determined gravimetrically, based on the ASTM E96-00 method [35]. The films were cut (discs with a diameter of 50 mm) and placed at the top of a permeation cell containing a desiccant. The cell was then placed in a humidity chamber at 23 °C and 50% RH. The cell was weighed over a 48-h period at intervals of at least 1 h. The calculations were carried out according to the ASTM method [35].

### 2.6. Opacity

Opacity of a material is an indication of light transmission: the higher the opacity, the lower the amount of light that passes through the material. For food packaging applications, this is usually done in the visible range (400–800 nm) [36]. In this work, the method used by Farhan and Hani [37] was adopted; a similar method was used by others [38,39,40,41], which was based on the ASTM method D1746-92 [42]. The apparent opacity of the samples was determined with a Shimadzu UV-Vis spectrophotometer (model 2600, Norwood, MA, USA), with UV Probe 2.43 software. A film rectangle measuring 400 mm long and 100 mm wide was adhered to the cuvette. Each sample was measured between 400 and 800 nm for the absorption value of visible light. Calibration for 100% transmittance was done using a cuvette without a sample. Film opacity was defined as the absorbance at 550 nm divided by the film thickness. Opacity was then expressed as absorbance units/mm (A-mm^−1^). Three measurements were made for each sample and averaged.

### 2.7. Thermal Analysis

Thermogravimetric analysis (TGA) was performed using a Q500 TGA instrument (TA Instruments, New Castle, DE, USA). Each sample (~5 mg) was weighed into a tared, open platinum TGA pan and measured in a nitrogen atmosphere by heating at 10 °C/min up to 600 °C. The data were analyzed for weight loss and derivative thermogravimetry (DTG) modes using the Universal Analysis software program (Version 4.5A) from TA Instruments (New Castle, DE, USA).

Differential scanning calorimetry (DSC) was conducted using a Q2000 DSC instrument (TA Instruments, New Castle, DE, USA). About 3 mg of each sample were weighed into an aluminum DSC pan, which was then hermetically sealed and analyzed under nitrogen atmosphere. The sample was equilibrated at 20 °C and heated at 5 °C/min up to 200 °C. DSC data were analyzed using the TA Instruments Universal Analysis software program (Version 4.5A).

### 2.8. Statistical Analysis

The data were shown as mean ± standard deviations and were also subjected to the analysis of variation (ANOVA) analysis in order to evaluate the significance of the difference between the means. Tukey’s test was used for comparison of the means, using the data analysis package in Microsoft Excel 2007. Values followed by the same subscript letter were not significantly different (*p* ≤ 0.05).

## 3. Results and Discussion

In this work, the layering approach was used to make blend films consisting of PLA and CSM layers. Five films were made with different amounts of CSM and PLA in the layers. Glycerol (0.3 g per gram of CSM, or 23%) was added to CSM as a plasticizer in all cases. The samples were (a) CSM film; (b) 90:10 CSM:PLA film; (c) 70:30 CSM:PLA film; (d) 50:50 CSM:PLA film; (e) PLA film. The thickness of each sample is summarized in Table 1. The five film samples showed thickness values in the range of 0.047–0.083 mm.

### 3.1. FT-IR-ATR Analysis

**Figure 1:**
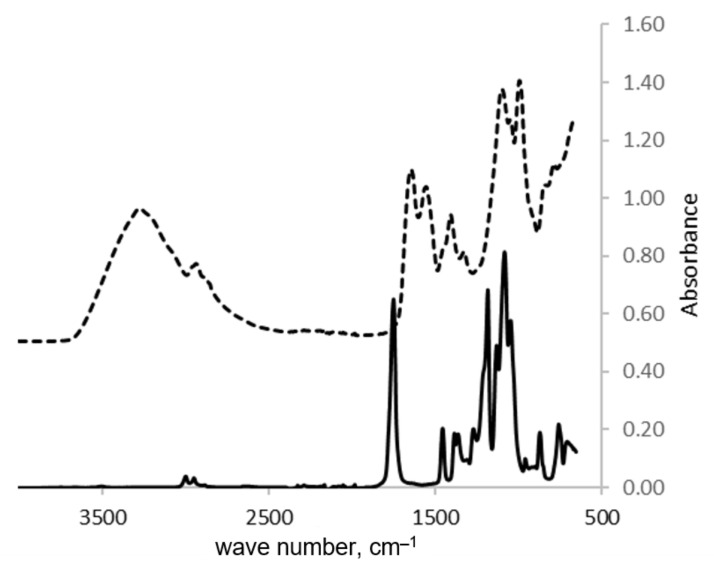
FT-IR-ATR spectra of the two surfaces of the 50:50 CSM:PLA bilayer film. Top spectrum is for the CSM surface, the bottom spectrum for the PLA surface.

First, it would be useful to confirm the chemical nature of the CSM/PLA bilayer film. Thus, the two surfaces of the CSM:PLA 50:50 film were examined by FT-IR-ATR. The spectra are given in Figure 1. The IR spectra of various cotton-related materials have been previously reported (e.g., [23,43,44,45]). The ATR spectrum of the CSM surface (Figure 1, top spectrum) showed a strong and broad band at 3700–3000 cm^−1^ due to the O-H and N-H stretching in carbohydrates, glycerol, adsorbed water, and protein. The smaller peaks at 2917 and 2863 cm^−1^ corresponded to CH_2_ asymmetrical and symmetrical stretching vibrations, respectively. The bands at 1628 and 1535 cm^−1^ corresponded to amide I band (mainly amide C=O stretching) and amide II band (N–H in-plane bending and C–N stretching). The weak amide III bands were observed near 1230 cm^−1^ and involved CN stretching and NH bending. The band at 1404 cm^−1^ corresponded to carboxylate vibrations in glutamic and aspartic acids [46]. The bands around 1040 cm^−1^ were related to the C-O stretching in hydroxy-amino acids (in the protein), in carbohydrates, and in glycerol. In general, these spectral features are consistent with the presence of protein, glycerol, and smaller amounts of carbohydrates in CSM.

The ATR spectrum of the PLA surface of the 50:50 CSM:PLA film is shown in Figure 1 (bottom spectrum). The IR spectra of PLA has been reported in a number of publications (e.g., [29,47,48]). The very small band at ca. 3500 cm^−1^ corresponded to the O-H stretching of the end OH unit. The C-H stretching in CH_3_ was given at about 2995 and 2947 cm^−1^. The ester C=O stretching was indicated by the 1748 cm^−1^ peak. The peaks at 1452 and 1372 cm^−1^ corresponded, respectively, to the asymmetric and symmetric deformation of CH_3_ C-H stretching. The peaks at 1186 and 1079 cm^−1^ were due to C-O stretching. In view of the IR data, the chemical structures of CSM and PLA in the CSM-PLA bilayer film have not changed, and there were no chemical reactions between CSM and PLA during the film preparation.

### 3.2. Mechanical Properties

The mechanical properties of the films are shown in the last three columns of Table 1. The CSM/glycerol film was found to have a YM of 87 MPa, TS of 2 MPa, and EB of 29%. These results are comparable to the values reported for soy protein by Gonzále and Igarzabal [29] and Salgado et al. [32]. In both of their papers on soy protein, the films were cast from aqueous solution of protein and glycerol, just like in this work. The soy protein films made by Gonzále and Igarzabal contained 20% glycerol and gave a YM of 22.8 MPa; TS of 1.1 MPa; and EB of 25% [29]. The soy protein films made by Salgado et al. contained 33% glycerol and gave a YM of 2.0 MPa; TS of 6.8 MPa; and EB of 54% [32]. The cottonseed protein film in this work contained 23% glycerol, and the TS and EB values are intermediate between the values reported by Gonzále and Igarzabal [29] and Salgado et al. [32]. However, the value for YM obtained in this work is somewhat higher than the soy protein films of both Gonzále and Igarzabal [29] and Salgado et al. [32].

In an earlier report on cottonseed protein isolate films made from formic acid solutions plasticized with 20% glycerol [23], better performance was reported, with YM 123 MPa, TS 9.5 MPa, and EB 189%. The different values reported in this work were due to the different solvents used (water versus formic acid) and the different sample types (cottonseed meal versus cottonseed protein isolate). There may also be some differences in the values due to the different testing methods used (ASTM D-882 in this work [34] versus ASTM D-638 in the earlier report [23]).

The bilayer CSM/PLA films gave reduced EB but much enhanced YM and TS values, relative to the CSM film. As expected, the PLA films showed the highest values of YM and TS. In a previous study of bilayer films of PLA and soy protein by Gonzále and Igarzabal [29], the insertion of the PLA layer also increased the mechanical properties of the bilayer films with respect to those of soy protein films. Thus, from the point of view of CSM, the use of the PLA layer significantly increases the tensile strength of the CSM film. From the point of view of PLA, the addition of the CSM layer may be an option to make the film somewhat more stretchable and also reduce the cost.

### 3.3. Water Vapor Permeation (WVP)

The WVP data for the CSM/PLA films are shown in Table 2. As expected, the CSM film showed the highest WVP. The addition of the PLA layer caused a reduction in WVP, with smaller values observed for thicker layers of PLA. This result is reasonable because PLA is hydrophobic and serves as a good barrier against moisture migration. Similar results were found for the PLA–soy protein bilayer films [29] and poly(3-hydroxybutyrate)–soy protein films [32], where the PLA improved the barrier properties of the soy protein.

### 3.4. Opacity

The opacity data for the same set of films are given in the last column in Table 2. The opacity for CSM film was about 20 A/mm, and for PLA film about 1.3 A/mm. The bilayer films showed intermediate values. The same trend was observed earlier for soy protein–PLA films [29], i.e., the protein film gave higher opacity values than the bilayer films. In a review by Nishinari et al. [49], it was noted that most vegetable protein gels are turbid, and the transparency increases with the protein content. It is known that vegetable proteins contain varying amounts of carbohydrate (including cellulose and hemicellulose), inorganics, lignin, oil, and phytochemicals [14,50,51], and these non-protein components may contribute to the observed opacity.

The high opacity is beneficial to food packaging because it can potentially reduce the light-induced reactions in the food items. However, in some applications, a film with a lower opacity may be desired if a consumer wants to observe the food item in the package prior to purchasing. Thus, these films with a range of opacity provide more options to be used as needed for a given application.

### 3.5. TGA Analysis

**Figure 2:**
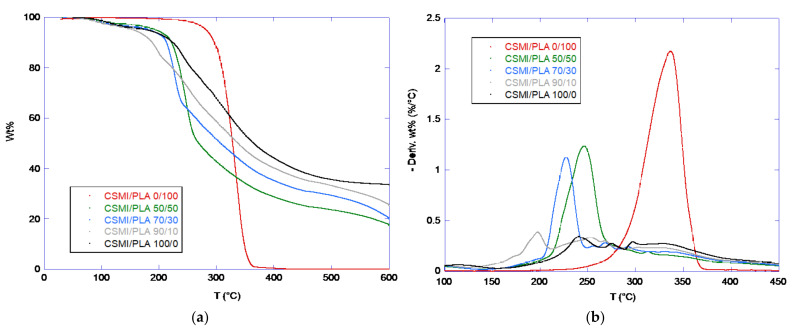
TGA (**a**) and DTG (**b**) data for the five bilayer film samples.

The TGA and DTG data for the five samples are shown in Figure 2. The weight loss below ca. 100 °C was due to water evaporation. The CSM appeared to degrade between ca. 200 and 400 °C and PLA at ca. 280–370 °C. For the bilayer films, each DTG curve showed a narrower peak due to PLA degradation and a broader peak for CSM degradation at 200–400 °C. However, the PLA degradation peak in the DTG curves shifted to lower temperatures as the PLA content decreased. Thus, the PLA degradation peak maximum occurred at ca. 245 °C for the CSM:PLA 50:50 sample, ca. 225 °C for the CSM:PLA 70:30 sample, and ca. 200 °C for the CSM:PLA 90:10 sample. Such a shift in PLA degradation seemed surprising, but a similar shift was found for the soy protein–PLA bilayer film, from 358 °C for PLA to 302 °C for the 60:40 soy protein: PLA bilayer film [29].

### 3.6. DSC Analysis

**Figure 3:**
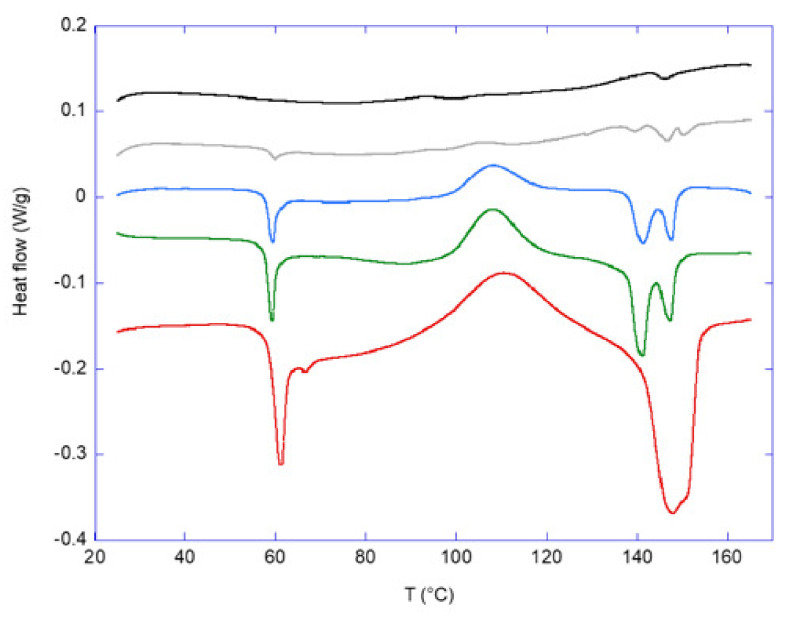
DSC data for the five film samples. From top to bottom, the plots correspond to 100: 0 CSM: PLA (black); 90: 10 CSM: PLA (grey); 70: 30 CSM: PLA (blue); 50: 50 CSM: PLA (green); and 0: 100 CSM: PLA (red).

The DSC curves for the five film samples are shown in Figure 3. The CSM/glycerol sample shows no strong peaks, and assignments are less certain. A small peak at ca. 90 °C was previously attributed to the denaturation of the protein [18,52]. The peak at 145 °C was also attributed to a denaturation process in earlier publications [16,18,53].

For the pure PLA sample (Figure 3), an endothermic peak showed up at ca. 60 °C, corresponding to the glass transition temperature (Tg); the broad exothermic peak at around 90–120 °C was due to cold crystallization, and the peaks at 140–155 °C were due to the melting of PLA. These features of the DSC of PLA were consistent with those reported before (e.g., [7,54]). Moreover, the melting of the pure PLA film gave a broad peak, but the bilayer films showed double melting peaks. As reported earlier [7], the double melting peaks might likely be associated with the presence of two crystal populations with different crystalline sizes or level of crystalline perfection, with the lower temperature peak corresponding to the melting of thinner lamella, less perfect crystals, and/or secondary crystals, and the higher temperature peak corresponding to the melting of thicker lamella and/or more perfect crystals [55]. If this was the case, the melting of PLA in the presence of the CSM surface (for the bilayer films) might possibly enhance this differential melting, thereby accentuating the double peaks.

## 4. Conclusions

For quite some time, the authors have been interested in using biodegradable polymers for food packaging [1]. PLA-cottonseed protein seems to be an attractive combination and has been explored in this work. (Glycerol was added to CSM as a plasticizer in all samples studied in this work.) The present results have shown the feasibility of producing the PLA–CSM bilayer films with varying thicknesses of the layers. From the point of view of PLA, the addition of the CSM layer reduces the cost and increases the opacity and stretchability of the film to some extent. From the point of view of cottonseed protein, previous studies of cottonseed protein as a bioplastic [15,16,17,18,24,53,56] have indicated its mediocre mechanical properties, which limit its usefulness as packaging films. The addition of the PLA layer has significantly increased the Young’s modulus and tensile strength of CSM, while reducing the elongation at break. Moreover, the WVP and the opacity of CSM have been reduced with the addition of the PLA layer. Thus, the PLA–CSM combination provides complementary benefits for the application of these films in food packaging applications.

It may be noted that both PLA and CSM are agro-based, sustainable, and commercially available. Their increased use in food packaging may help mitigate the current reliance on petroleum feedstock and reduce the environmental problems of plastic waste and microplastics. Our effort in this work, using bilayer CSM–PLA films, is a small step in this direction. Moreover, the new use of cottonseed meal has the additional advantage of adding value to the cotton industry and providing more economic benefits to cotton farmers [57].

## Figures and Tables

**Table 1:** Bilayer films of CSM and PLA: thickness, Young’s modulus (YM), tensile strength (TS), and elongation at break (EB).

Ratio CSM	Ratio PLA	Thickness * (mm)	YM * (MPa)	TS * (MPa)	EB * (%)
1	0	0.068 ± 0.006 ^a,b^	87 ± 8 ^e^	2 ± 0 ^d^	29 ± 6 ^a^
0.9	0.1	0.061 ± 0.018 ^b,c^	451 ± 55 ^d^	7 ± 1 ^d^	3 ± 1 ^b^
0.7	0.3	0.083 ± 0.001 ^a^	959 ± 43 ^c^	19 ± 1 ^c^	2 ± 1 ^b^
0.5	0.5	0.066 ± 0.003 ^a,b^	2125 ± 86 ^b^	41 ± 2 ^b^	2 ± 0 ^b^
0	1	0.047 ± 0.005 ^c^	3310 ± 58 ^a^	52 ± 8 ^a^	2 ± 0 ^b^

* The data in each column were subjected to analysis of variance using the Tukey test method; according to this analysis, the same superscript letter for two or more values indicates that these values are not significantly different at *p* = 0.05.

**Table 2:** Water vapor permeation (WVP) and opacity of bilayer films of CSM and PLA.

CSM Ratio	PLA Ratio	WVP * (g-m/kPa-d-m^2^)	Opacity * (A/mm)
1	0	0.00135 ± 0.00050 ^a^	20.32 ± 1.25 ^a^
0.9	0.1	0.00099 ± 0.00009 ^a,b^	14.85 ± 0.94 ^b^
0.7	0.3	0.00062 ± 0.00010 ^b^	13.95 ± 1.20 ^b^
0.5	0.5	0.00046 ± 0.00010 ^b^	7.42 ± 0.57 ^c^
0	1	0.00040 ± 0.00005 ^b^	1.26 ± 0.27 ^d^

* The data in each column were subjected to analysis of variance using the Tukey test method; according to this analysis, the same superscript letter for two or more values indicates that these values are not significantly different at *p* = 0.05.

## Data Availability

The data presented in this study are available on request from the corresponding authors.
